# A systematic review of the role of heat therapy for patients with intermittent claudication due to peripheral artery disease

**DOI:** 10.1177/1358863X20983475

**Published:** 2021-02-15

**Authors:** Amy E Harwood, Christopher JA Pugh, Charles J Steward, Campbell Menzies, C Doug Thake, Tom Cullen

**Affiliations:** 1 Centre for Sport, Exercise and Life Sciences, Faculty of Health and Life Sciences, Coventry University, Coventry, UK; 2 Cardiff School of Sport and Health Sciences, Cardiff Metropolitan University, Cardiff, UK

**Keywords:** heat therapy, intermittent claudication, peripheral artery disease (PAD)

## Abstract

Intermittent claudication (IC) is associated with impairments in quality of life and walking ability. Heat therapy is an emerging cardiovascular therapy, which may improve walking in patients with IC. We undertook a systematic review to establish current evidence for heat therapy for patients with IC. We searched five databases (Ovid Medline / PubMed, Embase, Scopus / Web of Science, Cochrane Library and Health Technology Assessment Databases). A total of 6751 records were screened with 76 full-text articles assessed for eligibility. We included three randomised control trials and three acute interventions. For chronic interventions, three different heat therapy interventions were used. The 6-minute walk distance significantly improved following whole-body immersion (*p* = 0.03; ES 0.94, 95% CI: 0.06–1.82), but not after Waon therapy or a water-perfused garment. Ankle–brachial pressure indices were significantly improved following whole-body immersion (*p* = 0.01; ES 1.10, 95% CI: 0.20–1.99) but not after other therapies. No form of heat therapy demonstrated statistical improvements in quality of life or brachial blood pressure. Acute interventions were characterised by large increases in limb blood flow and core temperature, and transient reductions in blood pressure post-heating. At present there are only three randomised controlled trials assessing heat therapy for patients with IC. Moreover, each of those randomised controlled trials utilised different heat therapies. There is also very limited study of the acute physiological responses to different heat therapy interventions in these populations. Future research should establish appropriate heat therapy protocols and implement more randomised trials to understand its effectiveness. **PROSPERO: CRD42020187941**

## Introduction

Lower limb peripheral artery disease (PAD) is caused by the build-up of atherosclerotic plaques in the arteries that supply blood to the legs and feet.^1^ It is estimated that over 236 million people have PAD worldwide.^2^ The classic symptomatic manifestation of PAD is intermittent claudication (IC), which is muscle pain or discomfort in the legs brought on by walking. This pain can be severely disabling and have huge impacts on walking duration.^3^ Functional capacity, balance^4^ and muscle strength are also frequently impaired.^5^

The current clinical recommendations for first-line treatment of IC include lifestyle changes, best medical therapy and a supervised exercise programme (SEP).^6,7^ Supervised exercise is effective in improving clinical indicators such as maximum walking distance and quality of life.^6,8^ However, the overall uptake, adherence^9^ and availability of programmes is low.^10,11^ In addition, some patients may not be able or willing to participate due to co-morbidities.^12^ Therefore, alternative or adjunctive therapies may be suitable to help improve symptoms and functional status in those who are unable to regularly exercise.

The chronic application of heat as a cardiovascular therapy has some convincing epidemiological evidence with habitual long-term sauna immersion being associated with substantial reductions in cardiovascular and all-cause mortality.^13,14^ Interestingly, the utilisation of heat therapy in patients with PAD is not a new concept with a report in the Lancet from 1927.^15^ However, the interest in heat therapy for clinical populations has increased in recent years. Despite the limited results owing to a small number of studies, there are some potentially promising effects of heat therapy for patients with IC. A small case-series study reported that improvements can be ascertained in pain-free walking distance following a chronic heat therapy intervention.^16^ In contrast, a recent randomised control trial seemed to demonstrate no benefit in walking distance for patients undergoing heat-therapy compared to a standard supervised exercise programme.^17^ Accordingly, there is currently conflicting evidence for the effectiveness of heat therapy for clinical populations.^18^

Therefore, the aim of this study was to conduct a systematic review on randomised controlled trials (RCTs) using heat therapy for patients with PAD. We aimed to identify whether heat therapy is effective at improving walking ability and lower limb haemodynamic compared with a control condition. A secondary aim was to assess the acute physiological responses to heat therapy in order to better understand the mechanisms that may underpin any potential beneficial effects of chronic interventions.

## Methods

This review was registered with The International Prospective Register of Systematic Reviews (PROSPERO) CRD42020187941 and adopted the PRISMA guidelines for RCTs assessing heat therapy for patients with PAD.^19^

### Search strategy

Five databases (Ovid Medline / PubMed, Embase, Scopus / Web of Science, Cochrane Library and Health Technology Assessment Databases) were searched from database inception to June 2020, with an additional search in October 2020. Only full-text articles published in English and relating to adults (over 18 years of age) were included. Titles and abstracts identified were independently interrogated for inclusion by two reviewers (AH & TC) and disagreement resolved by a third reviewer (CJS). The full texts of any potentially eligible articles were then independently screened against the inclusion / exclusion criteria. The reference lists of identified studies were also hand searched for other relevant articles. Search terms include the following: ‘peripheral vascular’ or ‘claudica*’ or ‘peripheral arter*’ AND ‘heat’ OR ‘hot temperature’ OR ‘heat therapy’ OR ‘therapeutics’ OR ‘waon therapy’.

### Inclusion criteria

We included RCTs that investigated any method of heat therapy in patients diagnosed with IC (Fontaine II / Rutherford stages 1–3). No limits were placed on the type of heat therapy, application of heat therapy, intervention frequency or duration.

### Risk of bias

The risk of bias in each study was assessed by two independent authors (AH & CJS) using the Cochrane Collaboration tool.^20^ This tool has three classification grades ‘high’, ‘low’ or ‘unclear’. Any disagreement was resolved by discussion with a third reviewer (TC) if required.

### Data extraction and management

Data extraction was undertaken using a standardised form and inputted into Microsoft Excel (2010; Microsoft, Redmond, WA, USA). For each study we extracted information on study characteristics, including: participants, sample size, inclusion/exclusion criteria, intervention components, outcome measures and main findings. The primary outcome from chronic intervention studies were maximum walking distance (MWD) or maximum walking time (MWT) as measured by either constant or graded load treadmill test or the 6-minute walk distance (6MWD). Secondary outcomes (where reported) included: pain-free walking distance (PFWD) or pain-free walking time (PFWT), as measured by a constant or graded load treadmill test, ankle–brachial pressure indices (ABPI), health-related quality of life, cardiovascular function (blood pressure) and physical activity levels. For acute interventions, we extracted peripheral blood flow, core and skin temperature, blood pressure, heart rate and circulating angiogenic and inflammatory signalling molecules.

### Data synthesis

A narrative synthesis regarding participant characteristics and study was completed for both chronic and acute heat therapy interventions. Effective heat therapy interventions were identified as those that induce a significantly greater change (*p* < 0.05) for at least one outcome, when compared to a control condition. For key outcome measures (where reported appropriately) the mean difference (MD) between pre- and post-group data and between-group effect sizes (ES) were calculated and adjusted using Hedges bias correction for small sample sizes.^21^ The ES were interpreted as small (0.20 to < 0.50), moderate (0.5 to < 0.80) and large (< 0.80).^20^ If necessary, study authors were contacted for more information to allow for computation of ES.

## Results

### Included studies

**Figure 1.**
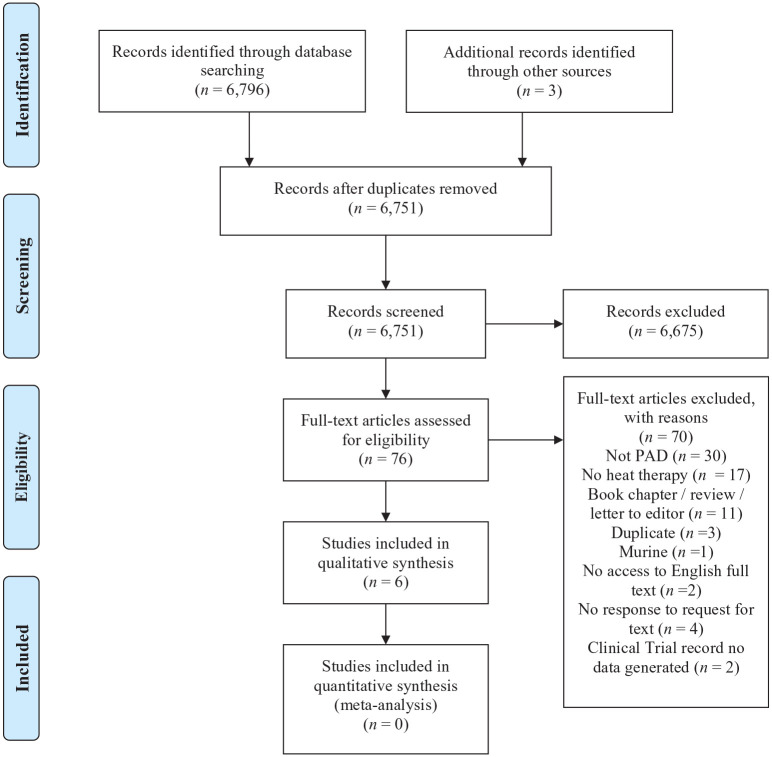
Preferred reporting items for systematic reviews and meta-analyses (PRISMA) flow diagram. PAD, peripheral artery disease.

The search yielded a total of 6751 records, of which, three RCTs and three acute interventions were included (Figure 1).

### Participants

For chronic interventions, 73 participants were included, with 37 completing a heat therapy intervention. All participants were reported to have mild–moderate claudication (Fontaine IIA/IIB), although it should be noted Akerman et al. included one patient in each group with ulcers.^17^ Mean age of included participants was 73 years and 76% were male. The large majority of patients included in the studies were hypertensive and diabetic. Medications included statins, aspirin, vasodilators, beta-blockers, calcium channel blockers and angiotensin-converting enzyme (ACE) inhibitors. Only one study specifically excluded women who were post-menopausal.^17^

For acute interventions, 49 participants were included with 33 completing a heat therapy intervention. All participants were reported to have mild–moderate claudication (Fontaine IIA/IIB). Mean age of included participants was 69 years of age, mostly male, and medications were similar to above.

### Included trials

Three studies met the inclusion criteria for intervention analysis.^17,22,23^ Akerman et al. compared heat therapy to a SEP, Shinsato et al. compared heat therapy to usual care (best medical therapy) and Monroe et al. compared heat therapy to a sham treatment. None of the heat therapy treatments (including duration, frequency and type) in the studies were the same (Table 1). The heat therapy treatments included whole-body immersion (up to shoulder height)^17^, Waon therapy (dry sauna)^22^ and lower-body water-perfused suit.^23^ Owing to these differences, we did not pool data for the purpose of a meta-analysis.

**Table 1.** Summary of RCT intervention findings.

Study	Sample	Descriptions of intervention	Outcome measures	Main findings
Akerman, 2019^17^ New Zealand Heat therapy (+ calisthenics) vs supervised exercise	**Total *n* = 22 (11 per group)** Individuals with mild–moderate IC, resting and exercise ABPI < 0.8 or > 0.2 drop, diagnostic duplex, > 45 years old Exclusions: Postmenopausal (not on HRT); bypass graft; aorta/iliac disease; type 1 diabetic; previous heat intolerance; unstable angina, MI or IHD (past 12 months); evidence in exercise test of undiagnosed IHD	Supervised hot immersion 3–5 × per week at 39°C for progressive duration (w1 = 20 min, w2 = 25 min, w3 = 30 min) to shoulder height. Followed by 15–30 min of warm clothing and calisthenic exercises (3 × per week): seated row, bicep curl, calf press, chest press, lateral raise, leg press, squat and tricep extension (room temp ~21°C). Contractions last for 1 min at 16–20 RPE, then 1 min rest (seating / standing). *Comparator*: SEP 2 × per week (30 min of self-paced walking and < 60 min of self-selected circuits). Each exercise lasted 3 min and exercise intensity self-selected. Total duration not reported.	6MWD PFWD ABPI QoL (SF-36) Physical activity BP and MAP FMD PWV VEGF Blood volume Serum ET-1 Adherence to treatment	Heat therapy: significant reduction in systolic BP (*p* = 0.049) No other parameters significantly different
Monroe, 2020^23^ USA Heat therapy vs sham	**Total *n* = 30 (15 per group)** Stable claudication > 6 months, ABPI < 0.9 Exclusions: uncontrolled diabetes, heart failure, COPD, CLI, ulcers; amputation; exercise-limiting comorbidities; recent < 3 revascularisations or planned; cancer; kidney disease; HIV; peripheral neuropathy; morbid obesity	Water-circulating garment that pumps hot water (47–50°C) round for 90 min to increase skin temperature to 40°C 3 × per week for 6 weeks. *Control*: Water-circulating garment that pumps thermoneutral water around leg (33°C) for 90 min 3 × per week for 6 weeks.	6MWD ABPI QoL BP and MAP Nitric oxide production Reactive hyperaemia CVC Serum ET-1 NIRS	Heat therapy: significant reduction in ‘Physical function’ (SF-36) (*p* = 0.018) and serum ET-1 (*p* = 0.03)
Shinsato, 2010^22^ Japan Heat therapy vs usual care	**Total *n* = 21 (11 in the intervention & 10 in control group)** Minimum duration of IC = 4 weeks, no evidence of improvement despite conventional therapies, ABPI < 0.75, plus imaging confirmation	Waon dry sauna at 60°C with no hydration pressure. Participants sat in sauna for 15 min and then underwent bed rest with a blanket to keep them warm for 30 min. Undertaken 5 × per week for 6 weeks. *Control*: Standard medical care.	6MWD ABPI VEGF Serum nitrate Leg pain CD34/GAPDH	Significant improvement in: leg pain (*p* < 0.05), 6MWD (*p* < 0.01), ABPI (*p* < 0.01) No statistical comparison to control

ABPI, ankle–brachial pressure index; BP, blood pressure; CD34/GAPDH, cluster of differentiation 34 / glyceraldehyde 3-phosphate dehydrogenase; CLI, critical limb ischemia; COPD, chronic obstructive pulmonary disease; CVC, cutaneous vascular conductance; ET-1, endothelin-1; FMD, flow mediated dilatation; HIV, human immune deficiency virus; HRT, hormone replacement therapy; IC, intermittent claudication; IHD, ischemic heart disease; MAP, mean arterial pressure; MI, myocardial infarction; 6MWD, 6-minute walk distance; NIRS, near-infrared spectroscopy; PFWD, pain-free walking distance; PWV, pulse wave velocity; QoL, quality of life; RCT, randomised control trial; RPE, rating of perceived exertion; SEP, supervised exercise programme; SF-36, Short-Form 36; VEGF, vascular endothelial growth factor.

Three acute intervention studies met the inclusion criteria.^24–26^ Thomas et al. undertook two waist-level immersion sessions (active: three 3-min bouts of plantar flexion vs passive),^26^ Neff et al. used a lower-body, water-perfused suit (two sessions of sham and heat therapy)^24^ and Pellinger et al. undertook two lower-limb heated immersions (15 min vs 45 min) and one sham immersion (Table 2).^25^

**Table 2.** Summary of acute intervention findings.

Study	Sample	Descriptions of intervention	Outcome measures	Main findings
Thomas, 2017^26^ New Zealand	**Total patients with PAD = 11** IIA / IIB IC; vascular diagnostic test; resting ABPI of < 0.7 in at least one leg; diagnostic duplex; age > 50 years and postmenopausal for females	Water immersion (42°C) up to the waist for 30 min	Popliteal and brachial artery blood flow and shear rate (anterograde, retrograde and total) BP and MAP Aural temperature HR PWV Muscle oxygenation	Significant improvement in popliteal and brachial artery shear rate and blood flow vs baseline (*p* < 0.0001). SBP, DBP and MAP significantly reduced during HWI (*p* < 0.001). HR significantly increased during HWI (*p* < 0.001). PWV decreased following HWI (*p* < 0.01). HWI significantly improved lower limb muscle oxygenation (*p* < 0.05).
Pellinger, 2019^25^ USA	**Total patients with PAD = 6** IIA / IIB IC; resting ABPI of < 0.9; BMI < 35; free of severe walking limitations due to comorbidities	Water immersion (40°C) up to a depth of 40 cm for 45 min	Popliteal artery blood flow (velocity and diameter) 6MWD HR BP	Limb blood flow and 6MWD significantly increased after HWI (*p* < 0.05).
Neff, 2016^24^ USA	**Total patients with PAD = 16** Resting ABPI < 0.9 in at least one leg; history of stable IC; significant aortoiliac or femoropopliteal disease determined through vascular imaging	Water-perfused suit up to the waist (48°C) for 90 min	BP and MAP Popliteal artery blood flow HR Core temp Skin temp VEGF MCP-1 IL-1Ra IL-1B, IL-6, IL-8, IL-10 TNF-α sTNFRII sVCAM-1, sICAM-1 NOx ET-1	Skin and core temperature significantly elevated vs control (*p* < 0.05). Marked increase in limb blood flow (*p* < 0.01) vs baseline. Heat therapy reduced systolic and diastolic BP (*p* < 0.05) vs control throughout the intervention. ET-1 reduced 30 min after heat therapy vs control. No change in other serum angiogenic, inflammatory and vasoactive mediators vs control.

ABPI, ankle–brachial pressure index; BMI, body mass index; BP, blood pressure; DBP, diastolic blood pressure; ET-1, endothelin-1; HR, heart rate; HWI, hot water immersion; IC, intermittent claudication; IL, interleukin; MAP, mean arterial pressure; MCP, monocyte chemoattractant protein-1; 6MWD, 6-minute walk distance; NOx, nitric oxide; PWV, pulse wave velocity; SBP, systolic blood pressure; sICAM-1, soluble intercellular adhesion molecule-1; sTNFRII, soluble tumour necrosis factor receptor type II; sVCAM-1, soluble vascular cell adhesion molecule-1; TNF-α, tumour necrosis factor-alpha; VEGF, vascular endothelial growth factor.

### Risk of bias

**Figure 2.**
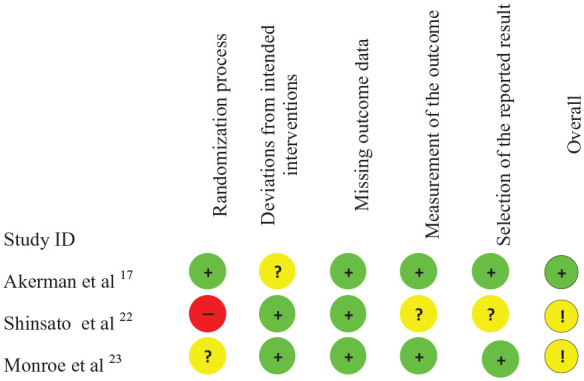
Risk of bias for included randomised controlled trials. Symbols indicate risk level: + indicates low risk; ? and ! indicate unclear risk; and - indicates high risk.

Risk of bias was only calculated for the chronic intervention studies. On average, studies generally had a low risk of bias, although some issues were identified (Figure 2). Owing to the limited number of studies, we did not report publication bias.

### Chronic interventions

#### Walking performance and clinical indicators

All three intervention RCTs included 6MWD as a measure of change in walking capacity. Results suggest that whole-body immersion^17^ and Waon sauna therapy^22^ increased the 6MWD, although no statistical comparison was made to controls. Means difference testing demonstrated this improvement was significant (*p* = 0.03; large ES 0.94, 95% CI: 0.06–1.82) in Akerman et al.^17^ Differences were not significant in Shinsato et al.,^22^ with a moderate ES (0.77, 95% CI: –0.12 to 1.66) (although it crosses zero) and a non-significant very small ES in Monroe et al. (0.16, 95% CI: –0.56 to 0.87), again crossing zero.^23^ Only Akerman et al. reported a change in PFWD post-intervention, with a moderate ES (0.53, 95% CI: –0.32 to 1.39), which was not significant between SEP and whole-body immersion.^17^

All three studies measured ABPI.^17,22,23^ Means difference testing demonstrated a statistically significant improvement in ABPI (*p* = 0.01; large ES 1.10, 95% CI: 0.20–1.99) favouring whole-body immersion compared to SEP.^17^ Shinsato et al. and Monroe et al. both had moderate ES, which were not statistically different when compared to best medical therapy and sham treatment, respectively (ES 0.50, 95% CI: –0.37 to 1.37 and ES 0.50, 95% CI: –0.14 to 1.32).^22,23^

#### Quality of life

Both Akerman et al. and Monroe et al. measured and reported quality of life using the Short-Form 36 (SF-36) tool.^17,23^ However, neither study reported the physical component summary or mental component summary and only presented results for individual components. For individual components, Monroe et al. reported a significant difference in the ‘physical function’ component favouring heat therapy (*p* = 0.018) compared to sham treatment after 6 weeks.^23^ Mean difference testing demonstrated that this was not significant with a small ES (0.25, 95% CI: –0.46 to 0.97). Akerman et al. reported a statistical difference between whole-body immersion and SEP in the ‘bodily pain’ component (*p* = 0.041).^17^ Mean difference testing demonstrated that this was not significant with a small ES (0.24, 95% CI: –0.60 to 1.08). Role emotion was significantly different (*p* = 0.05) between groups favouring heat therapy, with a large ES (0.86, 95% CI: –0.41 to 1.29), although it crosses zero.

#### Systemic haemodynamics

Both Akerman et al. and Monroe et al. reported blood pressure and mean arterial pressure responses to a heat therapy intervention.^17,23^ With regard to systolic blood pressure, Akerman et al. demonstrated that heat therapy reduced systolic blood pressure compared to SEP (moderate ES 0.52, 95% CI: 1.37–0.33) with no effect noted in Monroe et al. Diastolic blood pressure and mean arterial pressure was also reduced in Akerman et al. (small ES 0.41, 95% CI: –1.26 to 0.43 and moderate ES −0.59, 95% CI: −1.44 to 0.26).

### Acute interventions

#### Peripheral blood flow

All three acute studies included a measurement of popliteal flow pre and immediately post an acute heat therapy treatment. Results suggest that whole-body immersion^25,26^ and a lower-body heated garment^24^ increased limb blood flow. Pellinger et al. and Thomas et al. reported that blood flow in the popliteal artery was increased: large ES 4.13, 95% CI: 2.13–6.14 and large ES 1.53, 95% CI: 0.58–2.48, respectively. Neff et al. reported a statistically significant increase in popliteal artery blood flow (76%, *p* < 0.01); however, insufficient data were reported to calculate an ES.

#### Core and skin temperature

Both Thomas et al. and Neff et al. reported core temperature responses to an acute heat therapy treatment.^24,26^ Thomas et al. demonstrated an increase in core temperature by 1.8°C (aural measurement) at the end of the session (large ES 3.40, 95% CI: 2.09–4.70).^26^ Upon cessation of the intervention, Neff et al. demonstrated a significant increase in core temperature in comparison to the control condition (0.8°C, *p* < 0.01); however, insufficient data were reported to calculate an ES. Only Neff et al. reported changes in skin temperature – demonstrating an increase in skin temperature (~7.0°C) upon cessation of the heat therapy treatment (large ES 12.81, 95% CI: 9.6–16.03).^24^

#### Systemic haemodynamics

Both Thomas et al. and Neff et al. reported heart rate responses to an acute heat therapy treatment.^24,26^ Thomas et al. and Neff et al. reported increases in heart rate at the cessation of a single heat therapy treatment (large ES 1.89, 95% CI: 0.89–2.90 and large ES 5.83, 95% CI: 4.24–7.42, respectively). Both Thomas et al. and Neff et al. reported changes in blood pressure and mean arterial blood pressure at the cessation of a single session of heat therapy. Thomas et al. demonstrated a decrease in systolic (large ES 1.65, 95% CI: 0.68–2.62) and diastolic blood pressure (large ES 1.108, 95% CI: 0.18–2.43), while mean arterial pressure was also reduced (large ES 1.49, 95% CI: 0.54–2.43). Neff et al. also reported statistically significant decreases in systolic blood pressure (11 mmHg, *p* < 0.01), diastolic blood pressure (6 mmHg, *p* < 0.01) and mean arterial pressure (8 mmHg, *p* < 0.01); however, insufficient data were reported to calculate effect sizes or CIs.

#### Inflammatory and angiogenic markers

Only Neff et al. reported changes in circulating inflammatory and angiogenic markers. Endothelin-1 was significantly reduced (*p* = 0.026) 30 minutes after cessation of heat therapy treatment; unfortunately, insufficient data were reported to calculate the magnitude of this effect. Further to this, Neff et al. reported no changes in VEGF, MCP-1, IL-1Ra, IL-1B, IL-6, IL-8, IL-10, TNF-α, sTNFRI, sTNFRII, sVCAM-1, sICAM-1 and NOx (for definitions, please refer to Tables 1 and 2).^24^

## Discussion

The overall purpose of this systematic review was to identify whether heat therapy may be an effective therapy to improve walking distance and lower limb haemodynamics in patients with IC. The overall evidence from this review is limited owing to the low number of RCTs and heterogeneity between studies, and more studies are required before the efficacy of heat therapy for patients with PAD/IC can be clearly determined.

Encouragingly, despite the small number of studies, our results indicate that there may be some benefit to heat therapy; namely, whole-body water immersion and Waon therapy (but not a lower-body heated garment) regarding 6MWD. In particular, results from Akerman et al. demonstrated a significant large ES in comparison to a routine SEP. This represents a mean difference of 12 metres compared to a SEP and 47 metres compared to their baseline capacity,^17^ which is a large, minimal, clinically important difference.^27^ However, we note that patients in the heat therapy group also underwent resistance-band calisthenics-based exercises at least three times per week, so some of the observed improvement could have been mediated by participation in exercise. Nevertheless, Waon therapy also had a large, minimal, clinically important difference compared to best medical therapy, with patients increasing their 6MWD by 81 metres.^22^ This is potentially an important clinical finding for those patients who are unable or unwilling to exercise.

The improvements in walking capacity may be mediated by changes in peripheral haemodynamics. Indeed, whole-body immersion elicited a significant improvement in ABPI, which had a large effect size. However, this difference did not occur with the Waon therapy nor the water-perfused garment. This is an interesting finding, as ABPI does not seem to increase with supervised exercise programmes.^28,29^ It may be that heat therapy provides a greater stimulus for peripheral vasodilation, generating an angiogenic response. Indeed, Akerman et al. demonstrated significantly increased circulating vascular endothelial growth factor (VEGF) following chronic heat therapy; however, further investigations are required.^17^ Conversely, Neff et al. did not report any increases in VEGF, nor other angiogenic markers during an acute heating intervention with a lower-body heating garment.^24^ It remains to be seen whether whole-body heating, and the associated larger transient elevations in core temperature, induce angiogenic signalling. Despite these mixed results, chronic passive heating interventions have been shown to induce beneficial vascular adaptations in other sedentary adults.^30^ Further evidence is required in patients with PAD to determine angiogenic responses following chronic heat therapy.

Despite changes in 6MWD, none of the studies included demonstrated statistical improvements in quality of life, which is an important marker of disease severity in patients with PAD.^31^ Furthermore, studies did not calculate the physical and mental component summary scores, which are important distinct concepts. It may be likely that due to small sample numbers in studies they were not powered sufficiently to determine a change in quality of life. Future studies investigating heat therapy should calculate the physical and mental component summaries and use other common questionnaires, such as VascuQoL and the Walking Impairment Questionnaire. Furthermore, none of the included studies measured change in walking performance via a graded treadmill test. Graded treadmill protocols are a common outcome measurement to assess change in walking performance.^32^ They have the advantage of being conducted in a standardised setting (i.e. grade and speed of the treadmill are the same for each test)^32^ and have demonstrated good test-retest reliability.^33^ We would encourage future studies to utilise both a graded treadmill protocol and a 6-minute walk test to assess changes in walking distance.

Previous studies in other clinical populations^34^ and healthy older adults^35^ have demonstrated a reduction in systolic blood pressure following chronic heat therapy interventions. Reducing systolic and diastolic blood pressure may be of particular importance to those with PAD, given that hypertension is a major risk factor and a large proportion of patients are on antihypertensive medication.^2^ Despite this, no significant differences were reported between supervised exercise and whole-body heating immersion, nor lower-body water garment versus sham.^17,36^ However, large transient reductions in blood pressure were demonstrated in the acute interventions irrespective of the method of heating.^24,26^

We have demonstrated that at present there is no consensus as to the most appropriate method of heating, with each trial included in this review using a different method and frequency of stimulation. Accordingly, it is plausible that each respective heat therapy method may exert positive effects via different mechanisms. It appears at present that whole-body immersion may provide the largest stimulus and elicit the greatest benefit, although further research is required. Indeed, well-controlled acute studies are required to quantify the various physiological stimuli imposed by different methods of heating with a view to better understanding which interventions may provide most potential benefit when undertaken chronically. In this regard, it is clear that limb blood flow is increased dramatically irrespective of heating method. However, it remains difficult to provide a consensus for other potentially important physiological responses such as cardiovascular stress (i.e. increased cardiac output) and elevations in core and muscle temperature. In other cardiovascular populations, such as those with heart failure, evidence demonstrates improvement in left ventricular ejection fraction and quality of life regardless of the method of heat therapy,^37^ although the method of intervention is also highly variable in the reported literature. It may also demonstrate that those with more severe cardiovascular dysfunction may gain greater benefits, which has considerable clinical relevance, especially for those patients who are unable to tolerate an exercise programme.

Any intervention (or development of) should aim to be easy to deliver, practical and tolerable for patients. For example, whole-body immersion may be deliverable in the home-environment and could therefore ease patient burden to attend a centre. This could be of particular importance if patients are unwilling to attend a centre – in a similar manner to a home-based exercise programme. Furthermore, it may be suited to patients who have more advanced disease severity and cannot exercise, acting as an alternative or ‘gateway’ therapy. Further development of passive heating as an ‘alternative therapy’ should include careful consideration of the factors which negatively impact uptake and adherence. In the context of SEPs for PAD, this appears to be lack of access and tolerability.^10,12^ Future research should not only focus on developing interventions which are efficacious, but also those which encourage maximum uptake.

Finally, with regard to the safety of heat therapy, in all trials only one adverse event was reported in association with heating, which was described as skin irritation.^36^ However, further evidence is required to determine the safety of heat therapy and the risk of potential adverse events (such as hypotension and dizziness) both during and transiently following an acute bout of heat therapy.

### Limitations

The main limitation of our review is the low number of studies that were available for analysis. Alongside the low number of studies, the maximum number of patients included in any of the trials was 16.^36^ Despite this, we still found significant and clinically meaningful changes in some parameters with moderate to large effect sizes. Another limitation is that the modality of heat therapy was different across all trials, although we note that this appears to be similar in other cardiovascular conditions. Additionally, the comparator groups differed across all three intervention trials, and included sham, best medical therapy and an exercise programme. Finally, we were also unable to pool results for analysis and conduct a meta-analysis, so results should be interpreted with caution.

## Conclusion

This review demonstrated that at present there are only three RCTs assessing heat therapy for patients with IC. Moreover, each of those RCTs utilised different heat therapies. Only whole-body water immersion significantly improved 6-minute walk distance. The reason for the increased efficacy of whole-body heating is currently unclear and detailed acute studies will be required to understand the differences in physiological stimuli which underpin subsequent chronic adaptations. At present there appears to be some potential benefit to heat therapy as either an alternative or an adjunctive therapy for patients with IC. Future research should endeavour to establish appropriate heat therapy immersion protocols and implement more randomised trials in this cohort to understand its effectiveness.
